# Prevalence, Mortality, and Indicators of Health Care Supply—Association Analysis of Cardiovascular Diseases in Germany

**DOI:** 10.3389/fcvm.2018.00158

**Published:** 2018-10-30

**Authors:** Christina Dornquast, Stefan N. Willich, Thomas Reinhold

**Affiliations:** Institute for Social Medicine, Epidemiology and Health Economics, Charité – Universitätsmedizin, Berlin, Germany

**Keywords:** prevalence, mortality, cardiovascular disease, healthcare supply, association, regional differences

## Abstract

**Introduction:** There are regional differences in the morbidity of major cardiovascular disease between the 16 federal states of Germany. An association between the morbidity and the health care supply has been described in international studies. The aim of the present analysis was to examine the relationship between the prevalence or mortality of major cardiovascular disease and several key indicators of health care supply in Germany.

**Methods:** Life expectancy and the proportion of over 65-year old persons were included as characteristics to depict the general health. Indicators of health care supply were the number of general practitioners, internists, and cardiologists, number of internal medicine and cardiology beds, chest pain units (CPU), cardiac catheterization laboratories (CCL) and stroke units. In the form of an ecological analysis, we compared the cardiovascular disease prevalence and mortality with these indicators and performed a weighted linear regression.

**Results:** Regional variations between the federal states were found in general health and health care supply. The regression analysis yielded significant associations of the prevalence of major cardiovascular disease with the number of internal medicine hospital beds (β = 10.042, *p* = 0.045), cardiologists (β = −0.689, *p* = 0.031), and the number of residents per chest pain unit (β = 42,730, *p* = 0.036). Additionally, the relationship between cardiovascular mortality and also the number of residents per chest pain unit appeared to be significant (β = 4,962, *p* = 0.002). For all other indicators, no significant association was observed.

**Conclusions:** We detected regional differences in the general health and health care supply between the 16 German federal states as well as several significant associations between cardiovascular morbidity and health care supply indicators. Especially the decreasing number of cardiologists and rising number of residents per chest pain unit with an increasing prevalence of major cardiovascular disease should lead to a discussion about the structure of the Germany health care system, such as the needs-based planning mechanism of physicians. The results of this study may also aid in future development of other health care systems.

## Introduction

There are significant regional differences in the prevalence and mortality of major cardiovascular disease (CVD) between the 16 German federal states. In a previous study, we analyzed regional differences in the prevalence of major CVD using the German Health Update, a nationwide population-based study from the Robert Koch Institute (1). Several studies have identified a mutual dependence between the morbidity of a population and the supply of health care (2, 3). A German study from 2015 reported that regional health care availability has a positive effect on old age survival (4). These findings raise the question of whether there are differences in certain indicators of the health care supply in Germany in addition to the regional differences in major CVD morbidity?

Some international studies have examined the relationship between the disease burden of a population and the health care supply. Canadian scientists reported a discrepancy between physician supply and cardiac disease burden, in which the number of cardiac hospitalizations was inversely correlated with the number of cardiologists (5). A Japanese study similarly found a negative association between pediatrician density and mortality of under 5-year-olds (6). Several other studies have noted that greater primary care physician supply is associated with improvements in health outcomes such as obesity rates (7, 8).

The transferability of these international results to the context of the German health care system is limited and there has been less investigation into this field of research thus far. Correlation analyses of other topics have been performed in Germany but rarely for the relationship between disease burden and health care supply, especially in terms of CVD. A German study by Sundmacher and Busse investigated the impact of physician supply on avoidable cancer deaths (9). They showed a significant association between higher physician supply and lower avoidable cancer death rates for the selected cancer types. In addition, two studies have investigated the regional variation in the utilization of ambulatory services and hospital services (10, 11). They examined the influence of the Risk Structure Compensation Scheme (RSA) risk factor as a proxy for the average regional health status. Both studies reported an impact of this RSA risk factor on the utilization of health care services. Recently, studies in Germany have examined indicators of health care supply only and not in relation to morbidity. Some of these studies have reported an undersupply of physicians in several regions in Germany (12, 13).

In this paper, we focus on indicators of health care supply in the context of the CVD burden in Germany. The primary aim of the present study was to analyze the relationship between the prevalence and mortality of major CVD and several indicators of health care supply in Germany.

## Methods

### Study population and data sources

We performed a cross-sectional analysis in Germany with a regional distinction regarding the 16 German federal states. We used different data sources including a nationwide population-based study by the Robert Koch-Institute, official statistics from the Federal Statistical Office of Germany and different sources of claims data (Table 1).

**Table 1:** Data sources of health and health care supply indicators.

**Indicator (Year)**	**Data source**
Cardiovascular disease prevalence (2009–2012)	German Health Update (14)
Cardiovascular disease mortality (2009–2012)	Federal Statistical Office, Cause of death statistics, and Current population statistics (15)
Proportion of over 65-year old persons (2013)	Federal Statistical Office, Current population statistics (15)
Life expectancy (2013)	Federal Statistical Office, Period life tables (16)
Physician supply (2013)	German Medical Association, Statistics of physicians (17)
Hospital bed supply (2013)	Federal Statistical Office, Hospital statistics—basic data from hospitals and prevention or rehabilitation facilities (18)
Chest pain units (2015)	German Cardiac Society (19)
Cardiac catheterization laboratories (2015)	German Heart Foundation (20)
Stroke units (2016)	German Stroke Society (21)

### Health indicators

To characterize the cardiovascular health of the population in the 16 German federal states, we used the prevalence and mortality rates of major CVD identified in our previous study (1). For the prevalence, we combined the occurrence of four diseases (myocardial infarction, other manifestations of coronary heart disease, heart failure, stroke) to form a composite variable of major CVD. Mortality rates were calculated for the ICD-10 codes to which the four diseases were assigned. A detailed description of the calculation of prevalence and mortality is reported elsewhere (1). For the present analysis, we used the crude prevalence of major CVD to depict the actual disease burden in each federal state. By contrast, the mortality rates of major CVD were age- and sex-standardized (reference: Old European standard population) to account for the different age and sex distributions in the regions. By including two more indicators of general health, we examined whether the regional differences in the major CVD prevalence and mortality between the federal states of Germany were associated with differences in the demographic composition of the population. In the context of demographic change, the proportion of elderly individuals in a region could be an indicator of health care demand, especially as all types of CVD often occur in older ages. Furthermore, life expectancy at birth is regularly used as an indicator of the state of development of a region. Thus, we included the proportion of over 65-year old persons and the life expectancy of men and women in 2013 as indicators of general health in the federal states of Germany.

### Health care supply indicators

We included several health care supply indicators (as proposed by previous studies) on an aggregated level for the ecological analyses. We subdivided these indicators into general and cardiological health care supply indicators and acute medical care structure indicators. The general health care supply indicators comprised the number of outpatient general practitioners (GP), the number of internists without specialty and the number of beds in internal medicine wards in 2013 (5, 11, 22). In addition to the number of GPs, the two indicators of internal medicine physician and hospital bed supply were chosen because patients with major CVD are also treated in internal medicine departments as well as cardiology departments.

The cardiological health care supply indicators included the number of cardiologists and the number of beds in the cardiology hospital wards in 2013 (5, 22–24). These two indicators also functioned as characteristics of the medical treatment situation in the different federal states.

Finally, the indicators of acute medical care structure included the number of chest pain units (CPU), cardiac catheterization laboratories (CCL) and stroke units (SU) in 2016.

Regarding the number of hospital beds, it should be noted that a proportion of the inpatient cases had a residence in a different federal state than the one where they were treated. To consider that potential for overlapping health care, we reduced the number of beds in internal medicine as well as cardiology hospital wards in each federal state by the percentage of treated cases with a residence in other states.

Mortality and physician and hospital bed supply were expressed per 100,000 residents for both general and cardiological health care supply indicators. The indicators of acute medical care structure were presented as residents per each specific unit.

### Statistical analysis

To reach the primary aim of this study, we performed several ecological analyses. We descriptively compared the prevalence and mortality rates of major CVD with the two described indicators of general health in the 16 German federal states in relation to the national average. Possible relations between the major CVD prevalence or mortality and each health care supply indicator were examined by a weighted linear regression. The univariate associations were expressed in scatterplots using the trend line, the coefficient (β) and the resulting *p*-value of each regression. We used a weighted regression to account for the different population sizes in the federal states. Statistical analyses were performed using R version 3.0.2 (25).

## Results

### Characteristics of included indicators

The differences in major CVD prevalence and mortality have already been mentioned. Regarding the general health indicators, remarkable variations were observed for life expectancy of men and for the proportion of over 65-year old persons (Table 2). Furthermore, all indicators of the health care supply showed marked differences between federal states. For instance, the number of GPs varied between 38.0 and 54.9 per 100,000 residents, and the number of cardiologists ranged from 2.4 to 9.0 per 100,000 residents. The number of residents per SU differed from 99,072 to 399,437.

**Table 2:** Overview of the included indicators for the German federal states.

**Federal state**	**CVD prevalence**	Standardized CVD mortality^*^	**65+**	**Life expectancy men**	**Life expectancy women**	GPs^*^	Internists without specialty^*^	Beds internal medicine^*^	Cardiologists^*^	Beds cardiology^*^	**Residents per CPU**	**Residents per CCL**	**Residents per SU**
Baden-Württemberg	10.0%	116.5	19.7%	79.4	83.9	52.4	33.6	162.3	5.8	25.5	366,596	98,438	366,596
Bavaria	11.0%	131.7	19.8%	78.9	83.5	51.3	32.0	182.3	7.7	29.3	323,186	91,335	233,412
Berlin	11.5%	115.4	19.2%	77.8	83.0	39.0	52.9	180.7	5.8	44.1	285,152	100,642	228,122
Brandenburg	13.1%	152.4	22.8%	77.3	82.9	42.1	36.5	184.1	4.4	15.1	408,199	84,455	244,919
Bremen	11.8%	113.8	21.3%	76.9	82.3	40.0	49.7	210.3	6.2	15.7	328,696	93,913	328,696
Hamburg	10.2%	113.7	18.9%	78.2	83.0	45.9	47.1	172.6	9.0	36.2	249,477	67,167	174,634
Hesse	12.0%	120.4	20.1%	78.8	83.2	50.7	34.3	170.1	5.4	31.8	262,845	94,460	335,857
Mecklenburg-West Pomerania	12.7%	156.1	22.5%	76.5	82.8	46.7	35.2	210.7	4.8	28.2	399,126	79,825	177,389
Lower Saxony	12.9%	138.4	21.2%	77.8	82.8	47.2	31.0	171.0	5.4	27.2	354,116	102,507	354,116
North Rhine-Westphalia	12.2%	121.0	20.5%	77.8	82.6	38.0	48.9	213.7	3.2	30.5	358,609	91,046	270,336
Rhineland Palatinate	13.7%	138.7	20.6%	78.3	82.9	54.9	35.8	198.7	4.4	21.7	332,864	121,041	399,437
Saarland	10.5%	142.2	22.3%	77.2	82.2	48.6	51.3	204.4	2.4	34.6	330,239	99,072	99,072
Saxony	12.8%	157.9	24.7%	77.6	83.5	42.4	37.8	209.5	5.4	21.3	505,798	101,160	224,799
Saxony-Anhalt	15.8%	174.1	24.7%	76.2	82.5	40.1	40.8	230.1	3.3	21.7	748,192	106,885	374,096
Schleswig-Holstein	12.9%	125.8	22.3%	78.1	82.7	47.3	39.7	154.9	4.5	14.1	312,884	104,295	234,663
Thuringia	13.2%	149.7	23.7%	77.2	83.0	51.3	38.5	235.2	4.1	55.9	720,280	86,434	166,218
Germany	12.0%	131.0	20.6%	78.1	83.1	46.3	38.9	189.3	5.2	28.5	349,643	94,909	266,559

* *Per 100,000 residents. CVD, cardiovascular disease; GP, general practitioner; CPU, chest pain unit; CCL, cardiac catheterization laboratories; SU, stroke unit*.

### Descriptive analysis and comparison of health indicators

**Figure 1:**
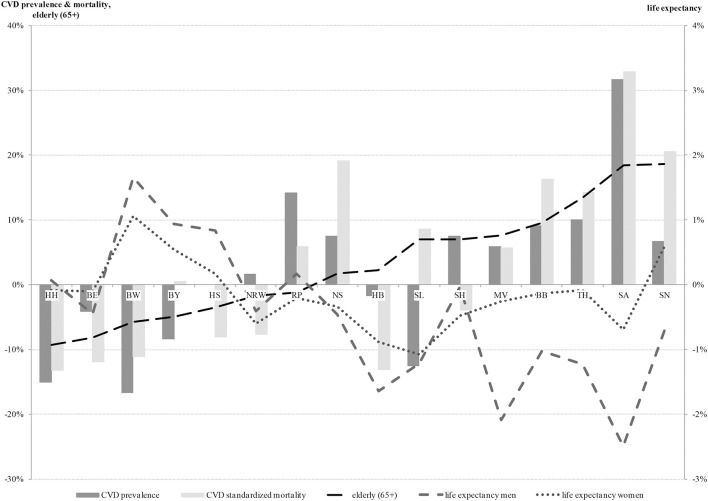
Health indicators in relation to the national average (0%) (ordered by proportion of the elderly). CVD, cardiovascular disease; HH, Hamburg; BE, Berlin; BW, Baden-Württemberg; BY, Bavaria; HS, Hesse; NRW, North Rhine-Westphalia; RP, Rhineland-Palatinate; NS, Lower Saxony; HB, Bremen; SL, Saarland; SH, Schleswig-Holstein; MV, Mecklenburg-West Pomerania; BB, Brandenburg; TH, Thuringia; SA, Saxony-Anhalt; SN, Saxony.

Different aspects of regional variations can be seen in Figure 1 which displays the descriptive analysis of all general health indicators in relation to the national average. An overview of all federal states revealed mostly rising rates of major CVD prevalence and mortality, with increasing proportions of over 65-year old persons. The distribution of over- and below-average values in relation to the national average was very similar for these indicators. Exceptions were observed in Rhineland Palatinate, Bremen and partly Saarland and Schleswig-Holstein. Concerning life expectancy, below-average values were found in particular in men in the new federal states (former East Germany), Bremen and Saarland. These federal states were also mostly characterized by over-average major CVD prevalence and mortality rates. In Baden-Württemberg, Bavaria and Hesse, the opposite in terms of over-average male and female life expectancy and below-average major CVD prevalence and mortality was observed.

### Regression analysis of CVD indicators and health care supply

**Figure 2:**
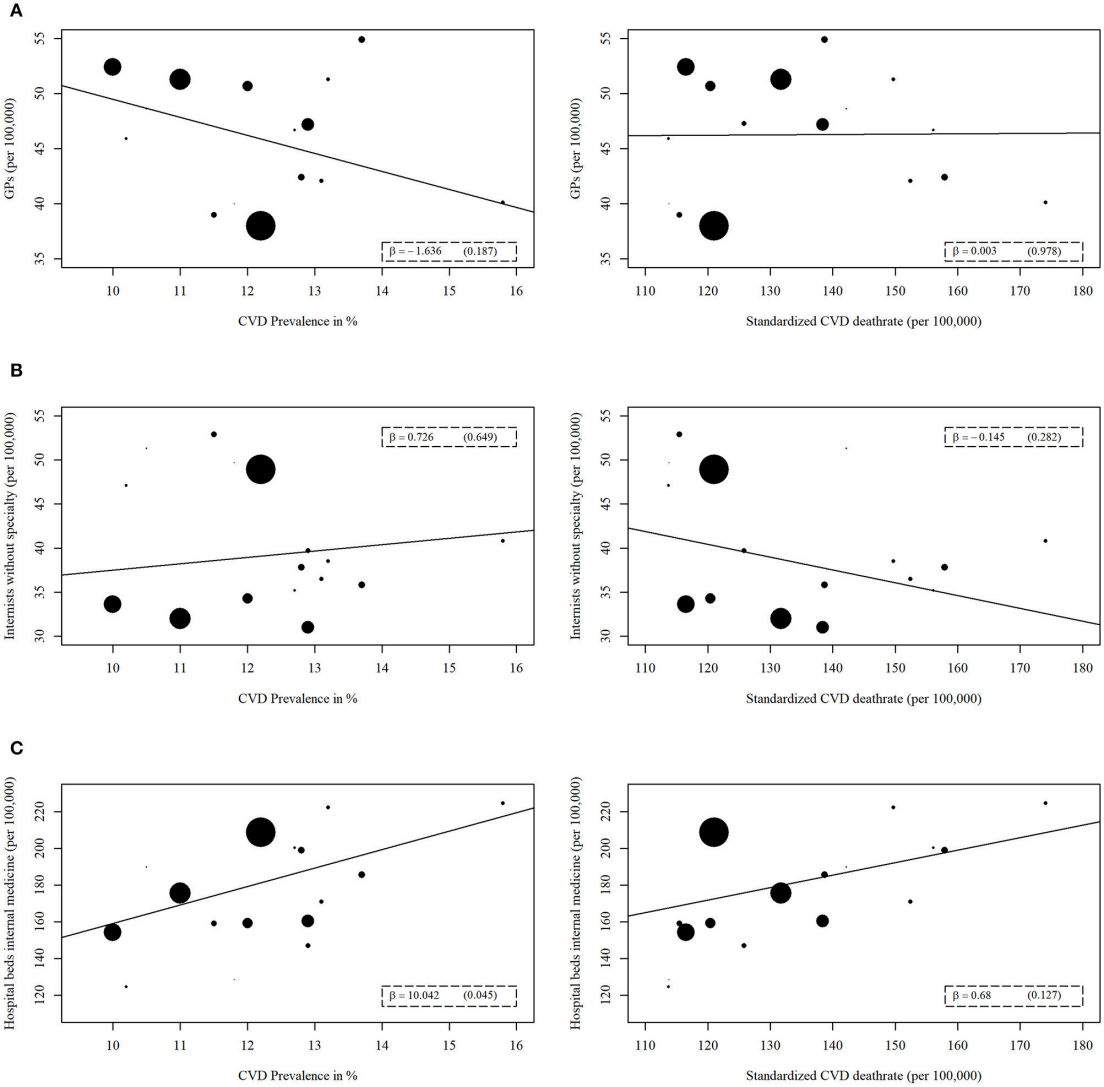
Regression analyses of general health care supply indicators with prevalence or mortality of major cardiovascular disease (CVD). The general health care supply indicators are **(A)** Number of general practitioners (GP), **(B)** Number of internists without specialty, **(C)** Number of hospitals beds for internal medicine. A weighted linear regression was done for every association. The weight refers to the population size of each federal state. The size of the data points reflects the different weights.

The regression analysis showed varying results for the relationships between the prevalence or mortality of major CVD and the included health care supply indicators. Regarding the general health care supply indicators (Figure 2), only one significant association was observed. The number of internal medicine hospital beds was positively correlated with the prevalence of major CVD (β = 10.042, *p* = 0.045).

**Figure 3:**
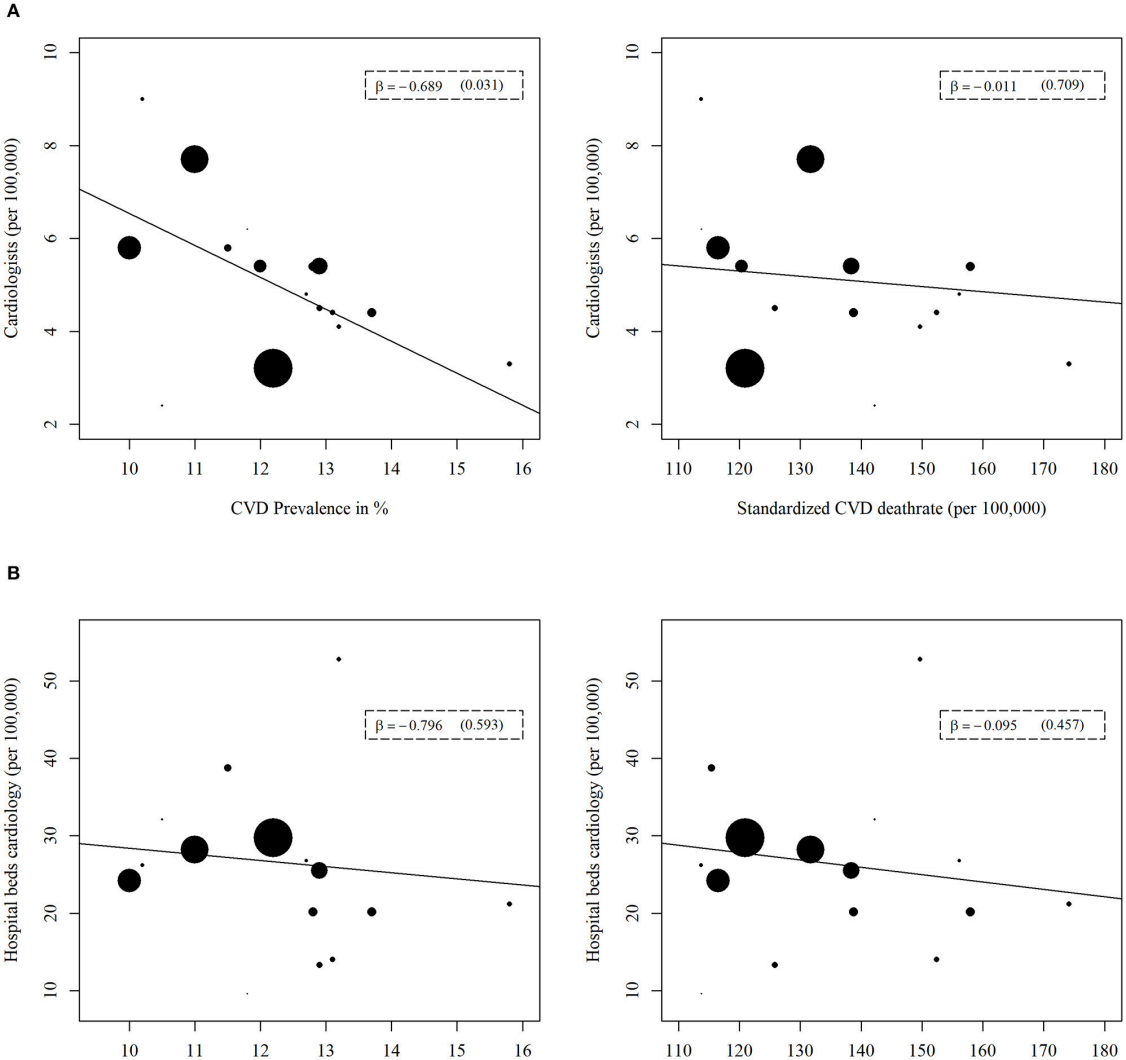
Regression analyses of cardiological health care supply indicators with prevalence or mortality of major cardiovascular disease (CVD). The cardiological health care supply indicators are **(A)** Number of cardiologists, **(B)** Number of hospital beds for cardiology. A weighted linear regression was done for every association. The weight refers to the population size of each federal state. The size of the data points reflects the different weights.

For the indicators of cardiological health care supply (Figure 3), similarly, one significant correlation was evident. Specifically, the number of cardiologists was the only variable that was inversely correlated with the prevalence of major CVD (β = −0.689, *p* = 0.031). For all remaining indicators, no significant correlation could be found in the context of general and cardiological health care supply.

**Figure 4:**
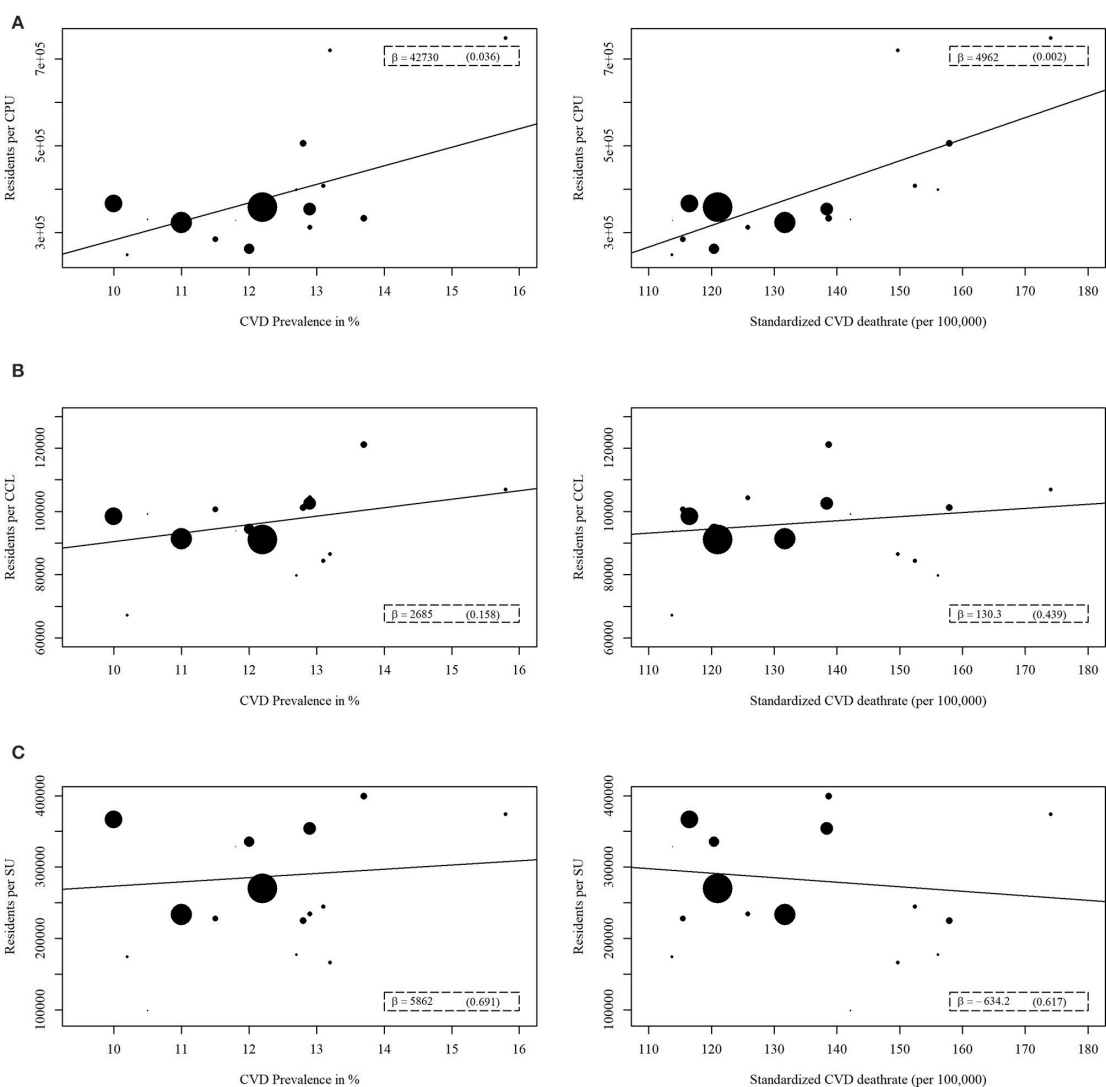
Regression analyses of acute medical care structure indicators with prevalence or mortality of major cardiovascular disease (CVD). The acute medical care structure indicators are **(A)** Number of residents per chest pain unit (CPU), **(B)** Number of residents per cardiac catheterization laboratories (CCL), **(C)** Number of residents per stroke unit (SU). A weighted linear regression was done for every association. The weight refers to the population size of each federal state. The size of the data points reflects the different weights.

Concerning the indicators of acute medical care structure, for both the prevalence and mortality of major CVD, significant results were evident for the association with the number of residents per CPU. This number was positively correlated with the prevalence (β = 42,730, *p* = 0.036) and mortality (β = 4,962, *p* = 0.002) of major CVD (Figure 4). The two indicators, number of residents per CCL or SU, were not significantly related to the prevalence or mortality of major CVD (Figure 4).

## Discussion

We identified regional differences in general health and health care supply between the German federal states with mostly worse positions for the eastern federal states. A weighted linear regression showed several significant results for the association between the health care supply indicators and major CVD prevalence as well as mortality. For prevalence of major CVD, associations were found for the relationship with the number of internal medicine hospital beds, the number of cardiologists and the number of residents per CPU. Additionally, the relation between major CVD mortality and the number of residents per CPU appeared to be significant. The other combinations showed no significant results.

These findings are consistent with those of previous studies. Alter and colleagues reported an inverse association of the number of cardiologists with the number of cardiac hospitalizations, based on a Pearson correlation coefficient of *r* = −0.34 (*p* = 0.01) (5). We found a similar relation between the number of cardiologists and the prevalence of major CVD in the 16 German federal states. Both studies demonstrated no associations between disease burden and the number of primary care providers or the number of GPs or internists without specialty. Regarding the relationship of the prevalence or mortality of major CVD with the number of residents per CPU, our data showed a significant positive association between these two variables. Several international studies support these findings. Furtado and colleagues reported a trend toward lower mortality after implementation of a CPU at an emergency department (24). Another study showed much higher mortality rates for patients with myocardial infarction if they were treated in a non-cardiology department instead of in a specialized coronary care unit (26). The relation between disease burden and number of hospital beds has been scarcely examined in other international studies.

However, the reasons underlying these achieved results remain unclear. In the context of regional analyses in particular, there are unobserved characteristics respectively confounding factors that might influence a specific development in the examined regions. Regarding the inverse correlation of major CVD prevalence with the number of cardiologists, previous studies have reported possible explanations. They have described that higher physician density could improve specific health outcomes (7, 8). With a higher density, each physician likely has to care for fewer patients, the patients have more alternatives and they have to travel shorter distances. An improvement in accessibility and medical care would be conceivable. This assumption is also supported by a study about the variations in the utilization of ambulatory services in Germany. The authors described that the so-called RSA risk factor, a proxy for the average regional health status of a population, influenced the number of ambulatory cases (11). Thus, contrary to the results of our analysis, with increasing morbidity, the supply of health care (e.g., the number of physicians) should also increase. A study by Ozegowski and Sundmacher also addressed this fact by analyzing the regional distribution of outpatient care providers in Germany (12). They showed an unequal distribution between the medical needs of the population and the existing capacities. The authors criticized that the German needs-based planning mechanism for physicians does not consider demography and morbidity of the population or accessibility of care (12). Moreover, this analysis reported a positive correlation between major CVD prevalence and the number of internal medicine beds. However, the relation between the prevalence of major CVD and the number of beds in cardiology wards showed no significant results. In this context, it could be proposed that patients with major CVD are not exclusively treated in cardiology wards at hospitals. Most likely, many of these patients receive inpatient treatment in internal medicine hospital wards. Additionally, the general bed space in internal medicine departments is much higher than that of cardiological hospital wards, which could also cause the examined relation. However, it should be stated, that regarding this positive relation between the number of internal medicine beds and the prevalence of CVD, a reverse causation is also conceivable. On the one hand it might be possible, that the number of internal medicine beds is higher in states with a higher prevalence because of the disease burden. On the other hand it might be possible, that due to the higher bed capacity more diagnoses occur in this field of diseases.

Finally, the positive relation of prevalence or mortality of major CVD with the number of residents per CPU should be discussed. This association is quite understandable because a fast and adequate treatment is highly important for patients with serious acute chest pain (e.g., myocardial infarction). CPUs provide specialized equipment and trained staff for these serious cases to ensure optimal care. Positive effects on various health outcomes such as mortality rates have been shown by international studies (23, 24, 26–29).

Regarding further unobserved explanations of the variations in prevalence and mortality of major CVD and the identified associations with health care supply indicators, a possible influence of social inequality between the federal states should not be disregarded. Socioeconomic status has been well examined as an explanatory factor for health differences and the utilization of health care supply in Germany (30, 31). Thus, socioeconomic status could have had a potential impact on our results.

To our knowledge, there are few studies that focus on comparing the morbidity of major CVD with the health care supply in Germany. Nonetheless, there are a number of limitations to our analysis. We described the associations between two variables with descriptive and regression analyses only. These kinds of ecological analyses cannot be used to do statements about causal relationships. The analysis or comparison was done on an aggregated level for the German federal states and not on an individual level for each hospital, let alone each patient. So, there could be an association on this aggregated level that does not have to apply to the individuals as well. Furthermore, our selection of health and health care supply indicators was somewhat arbitrary and we do not claim that all potential characteristics are encompassed. Possibly, these indicators reflect structures and incentives of the health care system rather than real correlations with the prevalence or mortality of major CVD. Also, there may be other confounding variables that could cover these issues and maybe function as indicators. These other factors might include the average travel time or distance to the nearest physician, the average arrival time of an emergency medical service, especially in rural areas or the proportion of guideline-compliant treatment. Further confounding factors could be the percentage of prescribed or taken cardiovascular preventive medication, doctor's proficiency and career, the organization of preventive programs and many others. Because of the lack of data on these potential indicators, they could not be considered as confounding factors in our analysis. Additionally, differences between urban and rural areas regarding physician density may be caused by overlapping catchment areas. For hospital beds, we tried to account for this by reducing the number of hospital beds by the percentage of treated patients who had residences in other regions.

## Conclusions

We observed regional differences in general health and health care supply between the 16 German federal states and several associations between the prevalence or mortality of major CVD and health care supply indicators. These results may indicate areas for improvement in the health care system of Germany. A change in the current needs-based planning system of physicians should be considered. The allocation of health care supply should be much more oriented toward the local morbidity and the medical needs of the population. The unobserved heterogeneity in the federal states indicates the need to conduct future longitudinal studies on this topic. These studies should examine the differences and correlations in morbidity and health care supply in smaller regional units, with the objective of establishing causal relationships. Furthermore, prospective studies on an individual level are desirable to confirm our findings.

## Data availability

The raw data supporting the conclusions of this manuscript will be made available by the authors, without undue reservation, to any qualified researcher.

## Author contributions

All authors contributed to the study concept. CD led the data preparation, analyses, and manuscript drafting and redrafting. SW was involved in revising the manuscript critically for important intellectual content. TR advised on and checked analyses performed by CD and revised the manuscript critically.

### Conflict of interest statement

The authors declare that the research was conducted in the absence of any commercial or financial relationships that could be construed as a potential conflict of interest.

## Abbreviations

CVD: Cardiovascular Disease
RSA: Risk Structure Compensation Scheme
GP: General Practitioner
CPU: Chest Pain Unit
CCL: Cardiac Catheterization Laboratories
SU: Stroke Unit.
